# *CDKN2B* Polymorphism Is Associated with Primary Open-Angle Glaucoma (POAG) in the Afro-Caribbean Population of Barbados, West Indies

**DOI:** 10.1371/journal.pone.0039278

**Published:** 2012-06-27

**Authors:** Dan Cao, Xiaodong Jiao, Xing Liu, Anselm Hennis, M. Cristina Leske, Barbara Nemesure, J. Fielding Hejtmancik

**Affiliations:** 1 Ophthalmic Genetics and Visual Function Branch, National Eye Institute, National Institutes of Health, Bethesda, Maryland, United States of America; 2 State Key Laboratory of Ophthalmology, Zhongshan Ophthalmic Center, Sun Yat-Sen University, Guangzhou, China; 3 Ministry of Health and University of the West Indies, Bridgetown, Barbados; 4 Department of Preventive Medicine, Stony Brook Medicine, Stony Brook, New York, United States of America; University of California, Irvine, United States of America

## Abstract

The purpose of this study was to confirm previously reported associations of common variants in or near *CDC7/TGFBR3*, *ZP4*, *SRBD1*, *ELOVL5*, *CAV1/CAV2*, *TLR4*, *CDKN2B*, *CDKN2B-AS1*, *ATOH7*, *PLXDC2*, *TMTC2*, *SIX1*, and *CARD10*, with primary open angle glaucoma (POAG) in the Afro-Caribbean population of Barbados, West Indies. A total of 437 unrelated subjects from the Barbados Family Study of Open Angle Glaucoma (BFSG), including 272 with POAG and 165 unaffected individuals were included in this study. Eighteen SNPs were genotyped by using the multiplex SNaPshot method. Allelic, genotypic and model-based (dominant, recessive, and additive) associations of the SNPs with POAG were analyzed using Chi-squared tests and logistic regression. SNP rs1063192 (near *CDKN2B*) was found to be significantly associated with POAG (allelic *P* = 0.0008, genotypic *P* = 0.0029), and the minor allele C of rs1063192 was protective against POAG (OR  = 0.39; 95%CI  = 0.22−0.69). Suggestive association was also noted for rs7916697 (near *ATHO7*, allelic P  = 0.0096, genotypic P = 0.01) with the minor allele being protective (OR  = 0.67; 95% CI  = 0.50−0.91), although this finding did not withstand correction for multiple testing. However, a significant interactive effect on POAG risk was identified between rs1063192 and rs7916697 (*P*-interaction  = 2.80×10^−5^). Individuals with the rs1063192 protective genotype CC or CT and also rs7916697 genotypes GG or GA show a significantly decreased risk of POAG (OR = 0.17, 95%CI: 0.07−0.41). Our study confirms the significant association between SNP rs1063192 (*CDKN2B*, previously shown to influence vertical cup-to-disc ratio and POAG at 9p21) and POAG in the Afro-Caribbean population of Barbados. The minor allele of rs1063192 interacts with that of rs7916697 (ATOH7)) to reduce POAG risk. Our results also suggest that rs1063912 is a common protective variant for POAG in populations of African as well as European descent.

## Introduction

Glaucoma is a complex disease characterized by degeneration of the optic nerve, often associated with increased intraocular pressure. It has been estimated that over 11.1 million people will be bilaterally blind from primary glaucoma by 2020 [1], making it the second leading cause of blindness worldwide. Primary open angle glaucoma (POAG) is more prevalent than primary angle closure glaucoma (PACG) in most populations. The prevalence of POAG increases markedly with age, and POAG tends to be more frequent in African derived populations [2], and especially in Afro-Caribbean populations [3], [4]. Progression of POAG is often painless and significant loss of vision can occur before patients seek ophthalmological care, so that identifying new ways to diagnose POAG early in its course or even predict its risk before visual loss occurs is of critical importance.

There is increasing evidence that POAG, and especially its endophenotypes, have a genetic component [5], [6], [7], [8]._ENREF_8 Familial aggregation of POAG has been documented in the general population [9], [10], and a high concordance of POAG between monozygotic twins has been observed, suggesting that genetic factors may play important roles in the pathogenesis of this disease [11], [12]. Linkage analyses have established three POAG susceptibility genes: *myocilin* (*MYOC*) [13], *optineurin* (*OPTN*) [14], and *WD repeat domain 36* (*WDR36*) [15]. However, taken together these genes only account for 3%-5% of sporadic POAG cases [8]. Therefore, candidate gene approaches and genome-wide association studies (GWAS) have been applied increasingly to investigate the molecular basis of this polygenic disease.

Recently a Japanese study using candidate gene methods found multiple SNPs in the *toll-like receptor 4* (*TLR4*) gene associated with the risk of normal tension glaucoma (NTG) [16], with an allelic *P* value of 0.0041 in the most highly associated SNP, rs7037117. In addition, six SNPs (rs547984, rs540782, rs693421, rs2499601, rs7081455 and rs7961953) comprising 3 different loci were modestly associated with POAG in a GWAS carried out on 1,575 Japanese subjects [17]. Another Japanese group carried out a separate GWAS, identifying *S1 RNA binding domain 1* (*SRBD1*) and *ELOVL fatty acid elongase* 5 (*ELOVL5*) as new susceptibility genes for both normal tension glaucoma (NTG) and POAG (with the most highly associated SNPs being rs3213787 in *SRBD1* and rs735860 in *ELOVL5*) [18]. In addition, a GWAS performed in an Icelandic population for POAG identified significant associations with SNPs between the *caveolin 1* (*CAV1*) and *caveolin 2* (*CAV2*) genes on chromosome 7q31, with the tightest association being to rs4236601 [19].

In part because the complex genetics of POAG have tended to resist direct analysis, attention has turned to a group of endophenotypes that are thought to contribute to or mark POAG, including cup-to-disc ratio and optic disc area [20]. Three groups have recently carried out GWAS using quantitative analytical approaches and identified several genomic regions showing associations with optic disc area and vertical cup-to-disc ratio (VCDR). Studies in Rotterdam and Singapore have identified significant association between optic disc area and alleles of SNPs near *cell division cycle 7 homolog* (*CDC7*), *transforming growth factor, beta receptor III* (*TGFBR3*, rs1192415), *caspase recruitment domain family, member 10* (*CARD10*, rs9607469), and *atonal homolog 7* (*ATOH7*, rs1900004 and rs7916697) [21], [22], [23]. In addition, a GWAS in two Australian twin cohorts identified an association between rs3858145 near the *ATOH7* gene and mean disc area [21]. The Rotterdam and Singapore study also identified two loci associated with VCDR in a GWAS, rs1063192 in the *cyclin-dependent kinase inhibitor 2B* (*CDKN2B*) gene on chromosome 9p21, and rs10483727 on chromosome 14q22.3-q23 near the *SIX homeobox 1* (*SIX1*) gene [24]. Some of the above genomic regions related to optic disc area or VCDR have been tested for association with POAG in Caucasians, yielding two SNPs significantly associated with POAG (rs1063192 and rs10483727 in the *CDKN2B* and *SIX* regions, respectively) [8], [24]. A later GWAS in Australians of European descent with advanced POAG found association with rs4977756, which is near the *CDKN2B antisense RNA 1* (*CDKN2B-AS1, ANRIL*) and in tight linkage disequilibrium with rs1063192. *CDKN2B-AS1* is adjacent to and regulates expression of the *CDKN2B* and *CDKN2A* genes [25].

However, all the GWAS described above were carried out in Caucasian or Asian populations, as were the subsequent confirmation studies. Replication, especially in other ethnic groups, is essential for establishing the credibility of a genotype –phenotype association and to avoid peculiarities of single populations. One such alternative population group consists of individuals of African ethnic origin, in which POAG tends to be more predominant and progresses more rapidly than in most other ethnic groups [3], [26], [27]. This study evaluated the association between POAG and these SNPs in the Afro-Caribbean population of Barbados, confirming association to rs1063192 near *CDKN2B* and demonstrating an interactive effect between rs1063192 and rs7916697 in the *ATOH7* region on POAG risk.

## Materials and Methods

### Study Population

This study was approved by the Institutional Review Boards of Stony Brook University Medical Center, the Barbados Ministry of Health and the National Institutes of Health Combined Neuroscience Institutional Review Boards. All participants provided written informed consent before participation in the study, and the study protocols conformed to the Declaration of Helsinki.

This investigation included 272 unrelated POAG patients and 165 unrelated controls from the Barbados Family Study of Glaucoma (BFSG) [9], [28], a follow-up study to the Barbados Eye Study [3] initiated in 1988. Controls were unaffected spouses recruited through the BFSG and unaffected individuals ascertained through the glaucoma clinic of the Queen Elizabeth Hospital, all of whom were unrelated. Briefly, first degree relatives of a POAG proband in the BES were invited to participate in the family study, and recruitment was further extended to first degree relatives of family members found to have POAG. Recruitment and diagnostic criteria for the BFSG were describe in detail by Nemesure et al. [29] and selection for the association study was detailed in Jiao et al. [28] and in Table 1.

**Table 1:** Diagnostic criteria for POAG.

Diagnostic test	Criteria
Visual field	
++	At least two abnormal visual field tests by Humphrey automated perimetry, as defined by computer-based objective criteria, i.e., positive results of hemimeridional analyses of threshold tests (C24-2 or C30-2 full-threshold program) and/or the presence of one or more absolute defects in the central 30 degrees (as tested with the C64 suprathreshold program; 3-zone strategy), with ophthalmologic interpretation as definite or suspect glaucomatous field loss
+	Less than two abnormal visual field tests or an inability to perform reliable automated perimetry (because of severe visual impairment or infirmity), with ophthalmologic interpretation as definite glaucomatous field loss
Optic disc	
++	At least two signs of optic disc damage present in fundus photographs and/or the ophthalmologic evaluation, including either a horizontal or vertical cup-disc ratio ≥0.7, narrowest remaining neuroretinal rim ≤0.1 disc diameters, notching, asymmetry in cup–disc ratios between eyes >0.2, or disc hemorrhages
+	Less than two signs of optic disc damage as described above (or unavailable photographs), with an ophthalmologic assessment or clinical record documenting definite glaucomatous optic nerve damage
Ophthalmologic examination	
++	Clinical diagnosis of definite POAG after examination by the study ophthalmologist to exclude other possible causes for disc and field changes
+	Previous POAG history and treatment and/or visual field and disc damage, although a definite POAG diagnosis was not made at the time of the BFSG visit (e.g., because of inconclusive or incomplete data); the study ophthalmologist confirmed the diagnosis through record review or rE-examination

++, most complete classification data; +, less complete but sufficient for classification.

Those with POAG had a minimum of at least one plus (+) sign in each of the three categories.

All participants received a comprehensive examination including anthropometric and blood pressure measurements, best corrected visual acuity based on the ETDRS chart, Humphrey perimetry with the C64 suprathreshold program, C24-2 and C30-2 full threshold programs, applanation tonometry, pupil dilation, lens grading with the lens opacities classification system II [30] by slit lamp, and color stereo fundus photographs of the disc and macula. All participants also received a comprehensive examination by the study’s ophthalmologists and an interview including medical, ocular, and family history information. A blood sample of 14 ml was obtained from each subject for DNA isolation and EBV transformation in some cases. Diagnosis of POAG required the presence of specific signs of glaucomatous optic nerve damage plus visual field defects, as well as an ophthalmologic evaluation (Table 1) [31]. Elevated IOP was not a criterion for POAG under this definition. Control individuals showed none of the signs of glaucoma listed in Table 1.

### Genotyping

Genomic DNA was prepared from peripheral blood lymphocytes and from transformed lymphocytes as described elsewhere [29]. All SNPs were genotyped by using the multiplex SNaPshot method (Applied Biosystems, Foster City, CA) as described previously [28]. Genotypes were determined using Genemapper4.0 software (Applied Biosystems, Foster City, CA).

### Statistical Analysis

Genetic association analyses were performed using the Golden Helix SNP and Variation Suite 7 (Golden Helix, Bozeman, MT, USA). All SNPs were analyzed for deviation from Hardy-Weinberg equilibrium (HWE) using the Chi-squared test. Allelic, genotypic and model-based (dominant, recessive and additive) associations of the SNPs with POAG were analyzed using the Chi-squared test. The odds ratio (OR) and 95% confidence interval (CI) were estimated using a 2 by 2 table. Gene-gene interactions were analyzed by including an interaction term in the logistic regression model based on a dominant model. Correction for multiple comparisons was carried out using a Bonferroni correction. For quantitative analysis of IOP the right eye was chosen arbitrarily as values from both eyes were quite similar (mean IOPod  =  22.8±6.9, IOPos  =  23.0±8.2, R^2^ = 0.66, p = 3.7×10^−30^). Only available IOP values in the absence of treatment were considered in this analysis (N = 113). In order to achieve normal distribution of the residuals, a log-linear transformation, log(iop+3), was used before analysis. While this transformation yielded residuals that could not be distinguished from a normal distribution with α = 0.1, transformations ranging from log(iop-3) to log(iop+7) yielded distributions meeting normality with α = 0.05.

## Results

A total of 437 unrelated subjects were recruited in the current study, including 272 POAG cases and 165 controls. The eighteen SNPs were genotyped in all subjects. The average ages of POAG cases is 67.6 and that of controls is 62 years old, and 55% of the cases are males while 31% of the controls are males (Table 2). However, the average ages for affected males and females (67.2 and 68.1, respectively), and unaffected males and females (62 and 62, respectively) are similar, as are the average IOP values for affected males and females (22.9 and 22.9, respectively) and unaffected males and females (15.7 and 16.2, respectively). All SNPs conformed to Hardy-Weinberg equilibrium (HWE) in both the case and control groups, except for rs7037117 and rs7961953, which showed minor deviations (combined HWE P = 0.005 and 0.001, respectively).

**Table 2:** Clinical and demographic characteristics of case and control individuals**.**

	POAG	controls
age (S.D.)	67.6 (11.8)	62.1 (12.4)
males	44.90%	68.60%
IOPod (S.D.) *	22.5 (7.0)	16.3 (3.5)
IOPos (S.D.)*	22.8 (8.3)	16.2 (3.8)

* mm Hg, includes patients receiving therapy.

In the basic allelic tests, rs1063192, a SNP that previously was shown to be associated with VCDR and POAG [8], [22], [24], was significantly associated with POAG in Barbadians (allelic *P* = 0.0008, Table 3) with a genotypic *P* = 0.0029. The C allele of SNP rs1063192 was more frequent in controls than in patients with POAG (9.4% vs. 3.9%; OR = 0.39, 95%CI: 0.22−0.69), indicating a protective effect of this minor allele. A second SNP, rs7916697, which has been shown to be related with optic disc area [23], also showed suggestive association with POAG in this population (allelic *P* = 0.0096, Table 3) with a corresponding genotypic *P* = 0.0131. However, after adjusting for multiple testing using a Bonferroni correction (corrected significant p = 0.0021: 0.05/24 for 18 allelic tests for individual SNPs shown in Table 3 and 3 model specific tests for each of 2 SNPs that showed significant or suggestive allelic p values and are shown in Table 4), only rs1063192 remained significant.

**Table 3:** Allelic tests of SNPs.

dbSNP ID	Chromosome	Nearest Gene	Minor Allele	MAF*(%) POAG	MAF*(%) Control	Chi-Squared *P* †	Odds Ratio (95% CI)	HWE‡ *P*
rs1192415	1	*CDC7/TGFBR3*	G	19.0	17.1	0.4802	1.14 (0.79–1.64)	0.957
rs547984	1	*ZP4*	T	31.7	30.5	0.7374	1.05 (0.77–1.44)	0.476
rs540782	1	*ZP4*	G	31.4	29.9	0.6570	1.07 (0.79–1.46)	0.548
rs693421	1	*ZP4*	A	31.3	29.1	0.5193	1.11 (0.81–1.52)	0.733
rs2499601	1	*ZP4*	G	27.1	29.5	0.4440	0.89 (0.66–1.20)	0.528
rs3213787	2	*SRBD1*	G	0.6	1.2	0.2882	0.45 (0.10–2.03)	0.865
rs735860	2	*ELOVL5*	T	4.6	6.2	0.3176	0.73 (0.39–1.36)	0.911
rs4236601	7	*CAV1/CAV2*	A	39.7	36.4	0.3332	1.15 (0.87–1.53)	0.258
rs7037117	9	*TLR4*	A	22.4	28.2	0.0571	0.73 (0.53–1.01)	0.005∧
rs1063192	9	*CDKN2B*	C	3.9	9.4	0.0008	0.39 (0.22–0.69)	0.640
rs4977756	9	*CDKN2B-AS1*	G	33.3	35.8	0.4507	0.89 (0.67–1.20)	0.328
rs7916697	10	*ATOH7*	G	24.4	32.4	0.0096	0.67 (0.50–0.91)	0.589
rs1900004	10	*ATOH7*	G	31.7	31.3	0.9076	1.02 (0.76–1.37)	0.269
rs3858145	10	*ATOH7*	G	41.1	41.5	0.9138	0.98 (0.74–1.31)	0.490
rs7081455	10	*PLXDC2*	G	30.4	29.6	0.8052	1.04 (0.77–1.41)	0.395
rs7961953	12	*TMTC2*	A	14.2	15.7	0.5559	0.89 (0.61–1.31)	0.001∧
rs10483727	14	*SIX1*	C	5.6	7.1	0.4151	0.77 (0.42–1.43)	0.604
rs9607469	22	*CARD10*	A	17.8	16.0	0.5096	1.13 (0.78–1.64)	0.611

* Minor allele frequencies.

† Chi-Squared *P*-value. The Bonferroni corrected significance level was 0.0021 ( = 0.05/24, for 18 allelic tests for individual SNPs and 3 model specific tests for each of 2 SNPs).

‡ Hardy–Weinberg equilibrium.

∧ For markers out of HWE association was also tested using the Cochran-Armitage trend test with no association being shown (P = 0.0573 for rs7037117 and P = 0.5561 for rs7961953).

**Table 4:** Association results of rs1063192 and rs7916697 in different models.

SNPs		rs1063192	(CDKN2B)		rs7916697	(ATOH7)
Phenotype		POAG (n = 272)	Controls (n = 165)		POAG (n = 271∧)	Controls (n = 165)
Allelic Analysis						
Alleles (%)	T*	523 (96.1%)	299 (90.6%)	A*	410 (76%)	223 (68%)
	C	21 (3.9%)	31 (9.4%)	G	132 (24%)	107 (32%)
HWE P		0.51	0.68		0.20	0.40
		P^1^	OR^2^ (95% CI)		P^1^	OR^2^ (95% CI)
Allelic P Values		0.0008	0.39 (0.22−0.69)		0.0096	0.67 (0.50−0.91)
Genotypic and Model Based Analysis						
Genotypes (%)	TT	251 (92%)	135 (82%)	AA	159 (59%)	73 (44%)
	TC	21 (8%)	29 (18%)	AG	92 (34%)	77 (47%)
	CC	0 (0%)	1 (1%)	GG	20 (7%)	15 (9%)
		P	OR (95% CI)		P	OR (95% CI)
Additive (Trend)		0.0007			0.0106	
Dominant		0.00096	0.38 (0.21−0.68)		0.0034	0.56 (0.38−0.83)
Recessive		0.6771	n/a^3^		0.9901	0.80 (0.40−1.60)

∧ One sample did not amplify when this marker was tested.

* risk allele, odds ratios are calculated for the minor (protective) allele and genotypes.

1 Chi-Squared *P*-value. The Bonferroni corrected significance level was 0.0021 ( = 0.05/24, for 18 allelic tests for individual SNPs and 3 model specific tests for each of 2 SNPs).

2 Odds ratio.

3 no individuals were homozygous for the risk allele.

Association results for rs1063192 and rs7916697 are similar when examined under dominant and additive models, although no significant association is seen for any marker under a recessive model (Table 4). rs1063192 Shows significant association with POAG in both dominant and additive models with *P* = 0.0010 and 0.0007 for dominant and additive models, respectively; the dominant OR  = 0.38 (0.21−0.68), Table 4. Again, rs7916697 is suggestively associated with POAG in both dominant and additive models (*P* = 0.0034 and 0.0106 for dominant and additive model respectively; Table 4), but this finding does not remain significant after a Bonferroni correction for multiple testing (adjusted corrected significance level  = 0.0028 for 18 tests). When alleles at these two markers were tested for association with IOP values, neither was significant (regression P = 0.39 and 0.99 for rs1063192 and rs7916697, respectively) and neither was significantly associated when IOP was analyzed as a dichotomous trait (allelic *Χ* ^2^ p = 0.54 and 0.27, respectively). In contrast, IOP was highly associated with POAG diagnosis (P = 1.5×10^−10^ and 4.4×10^−8^, for logistic and linear regression, respectively), although elevated IOP was not a diagnostic criterion for POAG in the BFSG (Table 1).

Of the two SNPs showing significant or suggestive association with POAG, rs1063192 is located near the overlapping CDKN2B and *CDKN2B-AS* genes and rs7916697 is located near *ATOH7* gene. We examined other SNPs in these regions for association with each other and with POAG. Alleles of the three chromosome 10 SNPs near *ATOH7* (rs190004, rs3858145 and rs7916697) did not show significant association with each other, nor did alleles of the two SNPs near *CDKN2B* on chromosome 9 (rs1063192 and rs4977756). Consistent with the lack of linkage disequilibrium between alleles of the SNPs themselves, haplotypes of SNPs in neither region show significant association with POAG (data not shown).

**Figure 1:**
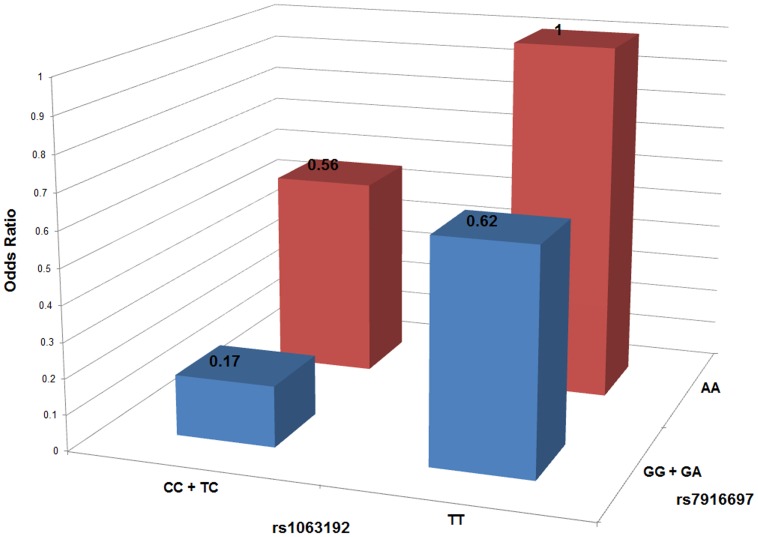
Interaction between rs1063192 and rs7916697. Logistic regression modeling showed that the joint effects between rs1063192 and rs7916697 were interactive (*P*-interaction = 2.80E−5).The joint odds ratios were estimated for combinations of protective genotypes of rs1063192 (in the dominant model) or rs7916697 (in the dominant model) compared with the combination of both risk genotypes (homozygous major alleles for both markers).

We then used logistic regression to investigate the joint two-locus effect of the two SNPs showing significant or suggestive association with POAG, rs1063192 and rs7916697. The results indicate a significant interaction effect between these two SNPs as risk factors for POAG, with comparison of the homozygous major alleles vs. a dominant model for the minor alleles of rs1063192 and rs7916697 giving a *P* interaction = 2.80E-5. Individuals with rs1063192 protective genotype CC or CT and also rs7916697 genotypes GG or GA have a significantly decreased risk of POAG (OR = 0.17, 95%CI: 0.07−0.41; Figure 1). In addition, comparison of the full interaction dominant model to rs7916697 alone yields a P = 0.00032, and comparison to rs1063192 alone results in a P = 0.0091, supporting the interactive effect of these two loci over that of each locus independently. This is consistent with the odds ratios obtained with a dominant model for each locus in the regression analysis against the homozygous main alleles for both markers (OR  = 0.56, 95% CI: 0.23−1.35 for rs1063192 and 0.62, 95% CI: 0.41−0.95 for rs7916697) and that for the combination of both rs1063192 and 7916697 together (OR  = 0.17, 95%CI: 0.07−0.41, Figure 1).

## Discussion

POAG is a complex disorder resulting from the combined interactions of multiple genes and environmental factors, so dissecting its genetic architecture can be simplified through the investigation of quantitative traits underlying disease risk. Intraocular pressure (IOP), optic disc parameters such as VCDR and optic disc area were reported to correlate genetically with POAG; therefore, searching for genes influencing these traits can aid in finding POAG genes even when association with POAG in the original studies is minimal [32]. In this study we included five SNPs associated with optic disc area (rs1192415 near *CDC7/TGFBR3*; rs9607469 near *CARD10*; rs190004, rs7916697 and rs3858145 near *ATOH7* ) and three SNPs related with VCDR (rs1063192 in *CDKN2B*; rs10483727 near *SIX1*; and rs4977756 near *CDKN2B-AS1*) to establish their roles in the pathogenesis of POAG in a population of African descent. Among the SNPs tested rs1063192, originally identified as a VCDR quantitative trait locus on 9q21 located in the 3′UTR for *CDKN2B* and within in an intron of CDKN2B-AS1 [22], was found to be significantly associated with POAG in the Afro-Caribbean population of Barbados. A second SNP, rs7916697 near *ATOH7* on chromosome 10 and previously related with optic disc area [21], [22], showed a nominal association with POAG although this did not withstand correction for multiple testing. In addition, risk alleles at these two SNPs were shown to interact, resulting in an increase in the risk for POAG with more than an additive effect, and the combination of both protective genotypes conferred a significantly decreased risk of POAG compared to that seen with either single SNP.

Association of rs1063192 and rs7916697 with POAG was not surprising in this investigation, since the definition of POAG in the Barbados Family Study of Glaucoma includes changes in the vertical cup to disc ratio and other disc abnormalities consistent with POAG as integral parts of the diagnostic criteria. Association of alleles at these two markers is also consistent with previous association studies in populations of European descent. A meta-analysis using data from six independent studies comprising 3,161 glaucoma cases and 42,837 controls found evidence for rs190004 (near *ATOH7*), rs1063192 (in *CDKN2B* and the overlapping gene *CDKN2B-AS1*) and rs10483727 (near *SIX1* gene) significantly associated with POAG [24]. Wiggs and her coworkers evaluated the association between SNPs associated with optic disc area and VCDR in a US Caucasian case/control sample and also identified associations between rs1063192 (in *CDKN2B*) and rs10483727 (near *SIX1*) and POAG [8]. rs1063192, The SNP related with VCDR, appears to be the only SNP consistently associated with POAG across the original and confirmation studies, including the current investigation. The C allele of rs1063192, which was reported to be significantly associated with a lower VCDR in a genome wide association study, had a protective effect to POAG in our Afro-Caribbean population as well as in the study by Fan et al [8].

A recent GWAS for POAG using a discovery cohort of 590 POAG cases with severe visual field loss and 3,956 controls also identified the POAG locus at CDKN2B/*CDKN2B-AS1*, with the most highly associated SNP being rs4977757 (*P* = 4.70×10^−9^) [25]. Interestingly, *t*he antisense RNA encoded by *CDKN2B-AS1* can regulate *CDKN2B*, with which its expression level is reciprocally related [33]. Since rs1063192, the most highly associated SNP found in this study, lies in the 3′UTR of *CDKN2B*, it was of interest to explore the relationship between rs1063192, rs4977756, and POAG. However, alleles of these two SNPs are in equilibrium in the BFSG study set (r^2^ = 0.053), and the haplotypes defined by these two SNPs do not show significant association with POAG risk (data not shown). However, since the haplotype block structure can vary significantly in different populations based on their history, lack of association with a single SNP does not rule out association of an entire region. Such exclusion would require exhaustive testing of tagging SNPs throughout the area.

SNP rs1063192, associated with VCDR, is located within the *CDKN2B* gene, which encodes a cyclin-dependent kinase that may regulate cell growth and has been implicated in the transforming growth factor beta (TGFbeta) signaling pathway. The *CDKN2B* region has been associated with other diseases such as gliomas, diabetes, and myocardial infarction [34], [35], [36], but the C allele of rs1063192, which reduces the risk of glaucoma, increases the risk for glioma. Currently the functional significance of rs1063192 is unclear. Since this SNP resides at the 3′-untranslated region (3′UTR) of *CDKN2B*, it is possible that the minor allele of this SNP may alter a regulatory element that affects the expression of *CDKN2B*, or it might simply be in linkage disequilibrium with a proximate true causal variant. In this regard, rs1063192 also lies within 10 kb of the *CDKN2A* promoter. Further investigation is needed to elucidate the exact causative variant(s) underlying these different disease associations.

Similarly, it is unclear why the C allele of rs1063192, which is protective against glaucoma, occurs at a much lower frequency in the Afro-Caribbean population of Barbados than in Caucasian populations, being intermediate between sub-Sahara African (near 0) and African Americans (about 0.13). While this might represent varying degrees of admixture, it is possible that it contributes to the higher incidence of POAG in these populations. One possible explanation for maintenance of these varying frequencies is that the onset of POAG is generally after the primary reproductive years so that it would not impact reproductive fitness greatly. In addition, if this allele increased risk for other diseases such as glioma [34] this could provide selective pressure to lower the allele frequency, especially if the risk was increased in the African relative to European populations.

SNP rs7916697, which plays a key role in controlling optic disc area, lies within *ATOH7*, a basic helix-loop-helix transcription factor required for retinal ganglion cell and optic nerve development [37]. Although this SNP is only marginally associated with POAG in the Barbados population, it shows significant interaction with SNP rs1063192 in decreasing POAG risk. Collectively, our results suggest that both common variants influencing normal variation in VCDR and optic disc area, affecting transcription and development, are involved in the development of POAG. Along with the lack of association of these SNPs with IOP, these results suggest that the associated genes might act through their effects on the retina and particularly in ganglion cells. This is consistent with the results recently reported by Wiggs et al. [38], and contrasts with the NRXN1 locus, which is highly associated with both POAG and IOP [28].

We were unable to confirm association to a number of other previously identified loci. The region harboring *CAV1* and *CAV2* genes on chromosome 7q31 has been reported to be associated with POAG in an Icelandic population (top SNP rs4236601, *P* = 5.0E−10) [19]. This was later confirmed by another group in a Caucasian US population in which the confirmation study found specific haplotypes located in the *CAV1/CAV2* intergenic region to be associated with the disease [39]. However, this association was not seen in a set of patients from Iowa [40], although the sample size in that study was smaller than that of Wiggs et al. [39]. We did not observe a positive association between rs4236601 and POAG in our Afro-Caribbean cohort. Our results suggest that this region may not be significantly related to POAG risk in populations of African descent. Nakano *et al.* demonstrated significant association of six SNPs with POAG that flanked genes on chromosomes 1 (*zona pellucida glycoprotein 4*; *ZP4*), 10 (*plexin domain containing 2*; *PLXDC2*), and 12 (*transmembrane and tetratricopeptide repeat containing 2*; *TMTC2*) in a Japanese population [17], but a subsequent replication study in an Indian population did not confirm this finding [41]. We were not able to find significant association to any of these six SNPs (rs547984, rs540782, rs693421, rs2499601, rs7081455 and rs7961953) with POAG in our cohort either (Table 3). We were also unable to replicate the association between *SRBD1*, *ELOVL5* and *TLR4* polymorphisms and POAG in our samples. This might be due to differences in the haplotype block structures of the various populations, to differences on the genes contributing to POAG risk in the populations, or perhaps simply to the relatively small size and correspondingly reduced power of the current study. In this regard, the Afro-Caribbean population of Barbados has a distinctive population history combining a founder effect from West Africa dating from the seventeenth century and limited influx of new populations since that time. Genetic associations generally are biologically more meaningful if they are replicated across different ethnic groups. In that regard, this study further supports the contributions of *CDKN2B and ATOH7*, or at least nearby loci, to POAG.

In summary, our study confirmed a significant association between SNP rs1063192 at 9p21 and POAG in the Afro-Caribbean population of Barbados and detected an interactive effect between rs1063192 and rs7916697 with POAG. The identification of the same susceptibility gene in both European and African descent populations helps to support the role of *CDKN2B/CDKN2B-AS1* in the pathogenesis of POAG. Further study of this gene and its related pathways will improve our understanding of POAG pathogenesis and its genetic susceptibility factors.
